# Regulation of Glial Cell Functions by PPAR-*γ* Natural and Synthetic Agonists

**DOI:** 10.1155/2008/864140

**Published:** 2008-04-22

**Authors:** Antonietta Bernardo, Luisa Minghetti

**Affiliations:** Department of Cell Biology and Neuroscience, Istituto Superiore di Sanità, 00161 Rome, Italy

## Abstract

In the recent years, the peroxisome proliferator-activated receptor-*γ* (PPAR-*γ*), a well known target for type II diabetes treatment, has received an increasing attention for its therapeutic potential in inflammatory and degenerative brain disorders. PPAR-*γ* agonists, which include naturally occurring compounds (such as long chain fatty acids and the cyclopentenone prostaglandin 15-deoxy Δ^12,14^ prostaglandin J_2_), and synthetic agonists (among which the thiazolidinediones and few nonsteroidal anti-inflammatory drugs) have shown anti-inflammatory and protective effects in several experimental models of Alzheimer's and Parkinson's diseases, amyotrophic lateral sclerosis, multiple sclerosis and stroke, as well as in few clinical studies. The pleiotropic effects of PPAR-*γ* agonists are likely to be mediated by several mechanisms involving anti-inflammatory activities on peripheral immune cells (macrophages and lymphocytes), as well as direct effects on neural cells including cerebral vascular endothelial cells, neurons, and glia. In the present article, we will review the recent findings supporting a major role for PPAR-*γ* agonists in controlling neuroinflammation and neurodegeneration through their activities on glial cells, with a particular emphasis on microglial cells as major macrophage population of the brain parenchyma and main actors in brain inflammation.

## 1. INTRODUCTION

The peroxisome proliferator-activated receptor-*γ* (PPAR-*γ*) belongs to the hormone nuclear receptor super family. It is a ligand-dependent transcription factor activated by both naturally occurring compounds, such as long chain fatty acids and the cyclopentenone prostaglandin 15-deoxy Δ^12,14^ prostaglandin J_2_ (15d-PGJ_2_), and synthetic agonists, including the thiazolidinediones (TZDs), and few nonsteroidal anti-inflammatory drugs (NSAIDs). Because of their role in the regulation of genes involved in lipid and carbohydrate metabolism, PPAR-*γ* and the other two isoforms PPAR-*α* and *δ*, deeply affect lipid homeostasis and insulin sensitivity [1–3]. The TZDs rosiglitazone (Avandia*®*), and pioglitazone (Actos*®*), introduced on the market in the early 1990s, are currently in clinical use to control blood glucose levels in subjects affected by type II diabetes.

In the last decade, accumulating evidence suggests that, besides diabetes and metabolic syndrome [4], PPAR-*γ* agonists have significant therapeutic potential in brain disorders. A large number of experimental studies and few clinical observations have suggested that PPAR-*γ* ligands may be successfully exploited to treat a wide range of neurological diseases, ranging from neurodegenerative diseases, to traumatic injuries, stroke, and demyelinating diseases, as recently reviewed by Heneka et al. [5]. In Alzheimer's disease (AD) transgenic mouse models, the TZD rosiglitazone attenuated learning and memory deficits [6], in line with its ability to promote cognitive preservation in patients with early AD [7, 8]. In amyotrophic lateral sclerosis (ALS) and Parkinson's disease animal models, the TZD pioglitazone ameliorated the disease symptoms [9, 10]. In rodent focal ischemia models, both pioglitazone and rosiglitazone decreased the infarct volume [11–13]. Furthermore, the natural agonist 15d-PGJ_2_ was shown to decrease the neurological deficits after experimental intracerebral hemorrhage [14] and its plasma levels in stroke patients were found directly correlated to the neurological outcome [15]. Rosiglitazone and pioglitazone decreased secondary neuronal damage, astrogliosis, microglial activation, myelin loss, and neuropathic pain in animal models of spinal cord injury while improving motor function recovery [16]. In experimental autoimmune encephalomyelitis (EAE), a well known model for autoimmune demyelinating diseases, synthetic, and natural PPAR-*γ* ligands—as well as some PPAR-*α* or *δ*agonists—have been reported to ameliorate clinical symptoms, to reduce expression of pro-inflammatory cytokines and chemokines, to decrease brain inflammation, demyelination and glial activation, and to delay the onset of disease [17–25]. More recently, promising results obtained in experimental models of ocular diseases have evidenced that PPAR-*γ* could be targeted to control inflammation and treat invalidating diseases such as diabetic retinopathy and optic neuritis, a demyelinating disease of the optic nerve frequently associated to multiple sclerosis (MS) (see for review [26]). Nonetheless, in spite of the amount of data on the therapeutic activities of PPAR agonists in EAE, clinical studies are still lacking and reports on their clinical use in MS or optic neuritis are still anecdotal [27]. Clinical trials are, however, in course with pioglitazone and rosiglitazone [5].

**Figure 1:**
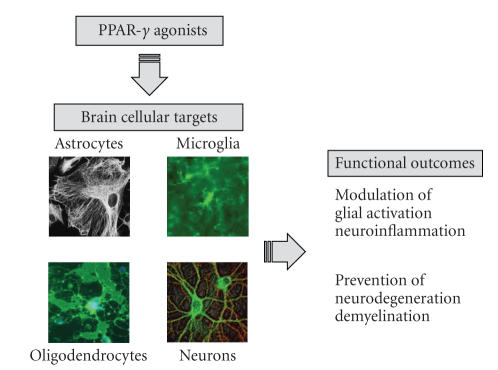
Cellular targets of PPAR-*γ* agonists in neurodegenerative diseases. PPAR-*γ* agonists can control neuroinflammation, neurodegeneration, and demyelination by effecting several cellular targets and by several direct and indirect mechanisms. PPAR-*γ* agonists can control glial activation, preventing a number of proinflammatory activities that can contribute to myelin/OL damage and neurotoxicity PPAR-*γ* agonists may also affect OLs and neurons, by preventing release inflammatory mediators and/or promote the synthesis of soluble factors or membrane-bound molecules that control glial activation.

The beneficial effects of PPAR-*γ* agonists in degenerative, inflammatory and traumatic brain pathologies are most likely mediated by several mechanisms, which may be disease-specific and involve both peripheral and central anti-inflammatory activities, by affecting crucial functions of peripheral (macrophages and/or lymphocytes) and central (microglial cells) immune cells. Besides microglia, PPAR-*γ* agonists can act on other neural cell types, including astrocytes, neurons, and oligodendrocytes (Figure 1).

Several of the beneficial effects of PPAR-*γ* result from its ability, once activated by specific ligand, to control the expression of proinflammatory genes, through the binding of specific sequences in their promoter regions—the peroxisome proliferator response elements (PPREs)—but also independently from its DNA-binding activity, by a mechanism termed transrepression, which have just begun to be elucidated [28]. In addition, some PPAR-*γ* ligands may exert specific activities independently from PPAR-*γ*. Among these, of great interest is the ability of a few TZDs to directly influence mitochondrial function by binding to target sites in mitochondria including the Complex I of the respiratory chain and the newly described protein mitoneet [29].

## 2. PPAR-*γ*: STRUCTURE, FUNCTIONS, AND AGONISTS

The PPAR-*γ* and the two closely related PPAR-*α* and PPAR-*δ* (also known as *β*, NUC-1, or FAAR) share a high homology, but differ for tissue distribution and ligand specificity [2]. PPAR-*α* is mainly expressed in tissues with high catabolic rates of fatty acids, such as the liver, muscle, and heart, whereas PPAR-*δ* shows a much wider distribution. PPAR-*γ* is highly expressed in adipose tissue and in cells of the immune system, including lymphocytes and macrophages. In the brain, PPAR-*γ* is expressed in several cell types including microglia, astrocytes, oligodendrocytes, and neurons.

PPAR-*γ* protein shows a remarkable conservation across species. Human and the murine PPAR-*γ* proteins show 95% identity at the amino acid level. The human PPAR-*γ* gene is located on chromosome 3 and generates at least three mRNA transcripts, PPAR-*γ*1, PPAR-*γ*2, and PPAR-*γ*3 [30–32]. PPAR-*γ*1 e PPAR-*γ*3 mRNAs encode for the same protein, while PPAR-*γ*2 mRNA gives rise to a protein containing 28 additional amino acids at the N-terminus.

At protein level, all three PPARs show a similar organization in five different functional domains, two of which—the DNA-binding domain (DBD) and the ligand-binding domain (LBD)—are the highly conserved [2]. The DBD contains the two zinc finger-like motifs that recognize the DNA target, and can be considered the hallmark of the nuclear receptor superfamily. The LBD conserves a common three-dimensional structure, which hosts a particularly large ligand-binding cavity, of which only 30–40% is occupied by the ligand. The relatively free nonspecific interaction between the cavity and the hydrophobic domains of the ligand explains the low ligand-specificity of PPARs. Nonetheless, the LBDs of the three PPAR isotypes have sufficiently divergent amino acid sequences to allow some ligand specificity.

Several unsaturated fatty acids bind to all three PPAR isoforms, whereas saturated fatty acids are in general poor PPAR ligands. However, given the relatively high concentration of lipids required for PPAR activation (in the micromolar or submicromolar concentration range), their “in vivo” role as PPAR ligands remains a controversial issue. Some arachidonic acid metabolites are more effective PPAR-*γ* ligands than the precursor. In particular, 15d-PGJ_2_, characterized by a reactive *α*,*β*-unsaturated ketone in the cyclopentenone ring, was the first PPAR-*γ* endogenous ligand, described in 1995 by two independent groups [33, 34].

The implication of PPAR-*γ* in several important metabolic and degenerative disorders, has strongly pushed the research of specific PPAR-*γ* agonists and antagonist (for review see [35]). A major group of synthetic PPAR-*γ* agonists is represented by the antidiabetic drugs TZDs, originally identified for their ability to improve the insulin sensitivity of diabetic animals. Pioglitazone and rosiglitazone belong to this group of high-affinity ligand. A different series of synthetic PPAR-*γ* ligands are derived by L-tyrosine GI262570, GW1929, and GW7845, which were developed on the basis of their activity on human PPAR-*γ* and are among the most potent PPAR-*γ* agonists, being active at low nanomolar concentrations.

In addition to these groups of ligands, several members of the heterogeneous NSAID family have been described as agonists for PPARs [35] and reference therein. In most cases, the doses required for PPAR-*γ* agonist activity are in the high micromolar range, thus largely exceeding those required for in vivo inhibition of cyclooxygenases (COXs), the main target of these drugs. Among NSAIDs, aspirin and acetaminophen (or paracetamol) lack of agonistic activity for any of the PPAR subtypes, whereas indomethacin, ibuprofen, and diclofenac are selective for the *γ*subtype. Recently, we have shown that the two nitric oxide (NO)-releasing derivative of flurbiprofen, HCT1026 and NXC 2216, were both able to activate PPAR-*γ* and induce its specific binding to a PPRE sequence [36, 37]. Few antagonists are also available, but their use is often limited by partial agonistic activity. The plasticizer biphenol A diglycidyl ether (BADGE) and the irreversible antagonist GW9662 are among the most widely used.

## 3. PPAR-*γ* AGONISTS AND OLIGODENDROCYTE BIOLOGY

**Figure 2:**
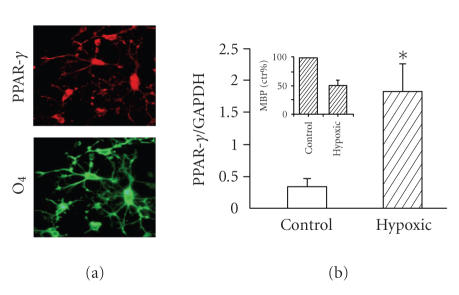
PPAR-*γ* expression in culture rat oligodendrocytes and in white matter (postnatal day 19) in rat model of global perinatal asphyxia. (a) Immunocytochemistry of rat OL progenitor cultures, prepared as previously described [40] for PPAR-*γ* (upper panel) and the OL marker O4 (lower panel). (b) Western blot analysis of white matter homogenates from rats at postnatal day 19 subjected to 20 minutes of perinatal asphyxia (hypoxic) and from controls, prepared as described in Piscopo et al. [48]. Inset show the decreased levels of MBP in hypoxic rats at pnd 19.

Oligodendrocytes (OLs) are the myelin-forming cells of the CNS. Their differentiation from precursor to mature cells occurs through a series of stages that can be defined by morphological and antigenic changes occurring in vivo as well as in culture systems [38]. During development and repair OLs extend elongated processes, forming multilamellar sheaths around neuronal axons. The formation, growth, and maintenance of the myelin sheath are prominent parts of neural development and nervous system function. As for OL maturation, myelin formation is a multistep process, involving recruitment to germination sites, proliferation of undifferentiated OL progenitors and their differentiation to mature OLs, producing myelin. Damage to OLs as a result of oxidative stress is considered a key pathogenetic pathway in several adult and infant human diseases. A substantial number of in vitro and in vivo studies has shown a maturation-dependent vulnerability to oxidative stress of the OL lineage [39–41], suggesting that OL progenitor is a key target for limit white matter damage and promote myelin repair [42]. Oligodendrocytes are major lipid producing cells, as required for myelin formation and maintenance. Given the role of PPARs in lipid metabolism it is conceivable that this group of nuclear receptor play a major role in OL differentiation and function. Although PPAR-*β*/*δ* has been long considered the PPAR type mainly expressed in OLs and involved in myelination [43, 44], recent findings support an important role for PPAR-*γ* activators in OL protection and differentiation. The first evidence for a role of PPAR-*γ* in OL differentiation was reported by Roth et al. [45]. By using the B12, oligodendrocyte-like cell line and primary cultures of spinal cord OL precursors, the authors first demonstrated that these cells expressed all three PPAR isoforms and found that natural and synthetic PPAR-*γ* agonists, but not other isoform activators, enhance process extension and cell maturation. These effects were blocked by the PPAR-*γ* antagonist GW9662. The maturation of pre-OLs was accompanied by enhanced expression of alkyl-dihydroxyacetone phosphate synthase (ADAPS), a peroxisomal enzyme required for the synthesis of plasmalogen, an etherphospholipid essential for myelin formation. These observations suggest that PPAR-*γ* mediated mechanisms may be important for OL differentiation and peroxisome functions. An important role for these organelles in maintaining OL and white matter integrity has been recently demonstrated in mutant mice characterized by the selective absence of functional peroxisomes from OLs [46]. In line with the proposed role of PPAR-*γ* in controlling OL differentiation and functions, we have recently confirmed the expression of PPAR-*γ* in highly purified rat OL cultures (Figure 2(a)). The level of expression is increased with the OL maturation in vitro (Bernardo et al., in preparation). In addition, we found an increased expression of PPAR-*γ* in white matter of young rats (post natal day 19) exposed to perinatal global asphyxia (Figure 2(b)). This model mimics some of the features of perinatal asphyxia, a major cause of immediate and delayed brain damage in the newborn [47, 48], and is characterized by early oxidative stress, delayed behavioral deficits, and alteration in myelin formation, as indicated by the strong reduction of myelin basic protein (MBP) expression (Figure 2(b)). Whether PPAR-*γ* over-expression is part of an adaptive response to the hypoxic condition aimed at restoring myelin formation or is part of an aberrant program leading behavioral impairment remain to be established.

In apparent contrast with the above findings, Xiang et al. [49], reported that the PPAR-*γ* natural ligand 15d-PGJ_2_, but not other PGs, induced apoptosis of OL precursor cell lines (mOP and CG4 cell lines). The toxic effect was developmental stage-dependent, being the undifferentiated mOP cells more susceptible than differentiated cells. In line with observations previously reported in microglia cultures [50], cell death was independent of the nuclear receptor PPAR-*γ*. Since the toxic effect of 15d-PGJ_2_ was prevented by preincubation of cell cultures with N-acetyl cysteine, a reducing agent and a precursor molecule for glutathione (GSH) synthesis, but not with free radical scavengers, the authors suggest that the underlying mechanism is related to oxidative stress due to depletion of GSH.

## 4. PPAR-*γ* AGONISTS AND ASTROCYTES

Astrocytes are most abundant glial cells in the CNS and crucial players in brain homeostasis. Among other functions, they provide metabolic support for neurons, uptake neurotransmitters such as glutamate, synthesize neurotrophic factors, and contribute to ion homeostasis (i.e., potassium uptake) and blood-brain barrier induction and maintenance [51]. In addition, astrocytes exert important roles also in brain inflammation and immunity, as they express several—though fewer than microglia—pattern-recognition receptors (PRRs) such as for example the Toll-like receptors, and release cytokines and chemokines that can trigger or amplify the local inflammatory response [52]. Similar to microglia, astrocytes rapidly react to a wide array of insults or damaging events. Reactive astrocytes, which are characterized by increased expression of glial fibrillary acidic protein (GFAP), a constituent of the intermediate filaments, are typical of most brain pathologies. Thus astrocytes represent an important target for anti-inflammatory and neuroprotective therapeutic strategies.

Astrocytes express PPAR-*γ* [53, 54], and accumulating evidence over the last ten years indicates that PPAR-*γ* agonists modulate astrocyte functions.

In rat cortical slices and cultured astrocytes, the TZD pioglitazone was found to significantly increase glucose consumption in time- and dose-dependent manners, through a mechanism independent of PPAR-*γ* and involving cAMP/PKA signaling [55]. Pioglitazone did not modify the expression of the glucose transporter GLUT-1, which is mainly expressed in glial and endothelial cells, but rather it increased glucose flux through pre-existing GLUT-1 protein. In addition, pioglitazone increased lactate production and release, induced mitochondrial membrane hyperpolarization, and protected astrocytes against hypoglycemia-induced cell death. On the basis of their studies, the authors suggest that TZDs modulate enzyme activities present within the mitochondrial membrane causing increased cytosolic pyruvate, resulting in greater lactate production. The inhibitory effect on mitochondrial function is compensated by an increase in anaerobic glycolysis allowing for continued ATP production. Eventually, the reduced intracellular glucose levels are replenished by glucose transport through the GLUT-1. At later times, mitochondrial respiration recovers, and accumulated ATP utilized to maintain and increase the membrane potential. Because hyperpolarization of the mitochondrial membrane is postulated to be protective, the net result of TZD treatment, at least in astrocytes, is protective and allows cells to withstand subsequent noxious stimuli [55]. Altogether, these results suggest that TZD-induced alteration of astrocyte metabolism and mitochondrial function could be beneficial in neurological conditions, in which glucose availability is reduced.

Another important mechanism by which PPAR-*γ* agonists could exert neuroprotection by influencing astrocyte functions is the enhancement of glutamate uptake. Romera et al. [56] reported that the PPAR-*γ* antagonists T0070907 prevented the ischemic preconditioning-induced (IPC) neuroprotection in neuronal-astrocytic cocultures subjected to oxygen-glucose deprivation (ODG) and reversed the inhibitory effect of IPC on OGD-induced glutamate release. In addition, rosiglitazone and the non-TZD agonist L-796,449 induced a concentration-dependent increase in glutamate transporter GLT-1 expression and [^3^H] glutamate uptake in rat astrocytes. In addition the authors identified six putative PPREs in the promoter region of GLT1/EAAT2 gene, suggesting GLT1/EAAT2 glutamate transporter is a novel PPAR-*γ* target gene [56]. Finally, 15d-PGJ_2_ remarkably increase the synthesis and release of neurotrophic factor nerve growth factor (NGF) in mouse primary astrocytes, which could further contribute to neuroprotection [57].

As mentioned above, activated astrocytes produce cytokines and other molecules involved in inflammatory response, which are thought to significantly contribute to brain damage. Such neurotoxic activities have been shown to be reduced by PPAR-*γ* agonists in several experimental paradigms. The two TZD compounds NP00111 and NP01138 were reported to inhibit the production of nitric oxide (NO), IL-6, and TNF-*α* as well as expression of the inducible enzymes iNOS and COX2 induced in LPS-stimulated astrocyte and microglial cultures [58]. Consistently with the described anti-inflammatory activities, the two compounds were neuroprotective in an animal model in which of brain damage is induced by kainic acid administration [59]. Both in vitro and in vivo effects were substantially attenuated by cotreatment with the PPAR-*γ* antagonist GW9662, supporting the involvement of PPAR-*γ* activation.

In contrast to the above described TZDs, the natural ligand 15d-PGJ_2_ prevented the IL-1*β*-induced COX-2 mRNA accumulation in human astrocytes, through a PPAR*γ*-independent mechanism [60]. Similarly, Lennon and colleagues [61] showed that ciglitazone and 15d-PGJ_2_ activated the MAP kinase cascades (Erk, Jnk, and p38 MAP kinase) in astrocytes by a PPAR-*γ* independent mechanism, which required the presence of ROS. Again, independently of PPAR-*γ*, 15d-PGJ_2_ and rosiglitazone reduced the phosphorylation of signal transducers and activators of transcription (STAT) 1 and 3 as well as Janus kinase 1 (JAK1) and JAK2 in activated astrocytes and microglia [62].

Recently, Xu and Drew [63] extended the analysis of the anti-inflammatory activity of PPAR-ligands to other inflammatory mediators belonging to the IL-12 family of cytokines. They found that in primary astrocytes, LPS induced the production of IL-12p40, IL-23, and IL-27p28 proteins, which was significantly reduced in the presence of 15d-PGJ_2_. Since these cytokines play critical roles in the differentiation of T helper (Th) 1 and Th17 cells and are likely to contribute to the development of multiple sclerosis, this observation further support the potential role of PPAR-*γ* agonists in MS treatment [5, 64].

In line with the beneficial effect of PPAR-*γ* agonists in experimental models of inflammatory diseases, PPAR-*γ* has also been involved in anti-inflammatory functions of IL-4, a Th2 type cytokine, which plays an important role in controlling Th1 cell responses and resolution of inflammation. Paintlia et al. [65] demonstrated that the inhibition of iNOS expression and the increase of survival of differentiating OPs induced by IL-4 in inflammatory cytokine-stimulated mixed cultures are mediated by PPAR-*γ* activation. In support of their conclusions, the authors describe a coordinate increase in the expression of both PPAR-*γ* and its natural ligand-producing enzyme 12/15-lipoxygenase (12/15-LOX) in IL-4-treated glial cells and show that IL-4-induced PPAR-*γ* activation antagonizes NF-*κ*B transactivation in inflammatory cytokine-stimulated astrocytes. A similar upregulation of PPAR-*γ* by IL-4 was demonstrated in cultured microglial cells [66]. To link between IL-4 and PPAR-*γ* is completed by the observation that the anti-inflammatory activity of the TZD troglitazone was mediated by its ability to increase IL-4 expression in glial cultures [67].

Astrocytes recognize and react to several pathogens through their repertoire of PPRs [52]. In a recent study, 15d-PGJ_2_ and ciglitazone suppress the production of IL-1*β* and NO in Staphylococcus aureus-stimulated astrocytes [68]. Interestingly, 15d-PGJ_2_ attenuated TLR2 expression, the PPR recognizing Staphylococcus aureus. Importantly, 15d-PGJ_2_ and ciglitazone were still capable of inhibiting the release of proinflammatory mediators induced by Staphylococcus aureus in PPAR-*γ*-deficient astrocytes, supporting PPAR-*γ*-independent effects of these compounds. In another study, 15d-PGJ_2_ significantly attenuated astrocyte reaction to mycotoxin ochratoxin A (OTA), a widespread food contaminant that accumulates in the brain. At noncytotoxic concentrations, OTA down-regulated GFAP expression while it upregulated vimentin. Interestingly, OTA increased PPAR-*γ* expression, possibly increasing the susceptibility of OTA-exposed cells to PPAR-*γ* agonist treatment [69].

## 5. PPAR-*γ* AGONISTS AND MICROGLIAL CELLS

Microglia derive from myeloid precursors that enter the developing CNS to become the major population of brain resident macrophages. Under physiological conditions, microglia show a ramified morphology and the absence of cell-surface and cytoplasmic molecules typically associated with other tissue macrophages. In this quiescent or “resting” state microglia are able to “sense” subtle environmental changes to which they rapidly react [70]. Although our knowledge on microglial in physiological conditions is still limited, using transgenic mice showing specific expression of enhanced green fluorescent protein in microglia and in vivo two-photon microscopy, it was shown that “resting” microglia constantly survey the surrounding microenvironment with extremely motile processes and protrusions [71]. Once activated, microglia rapidly undergo morphological changes, characterized by cell body enlargement, loss of ramified processes, and upregulation of cell-surface and/or cytoplasmic antigens. In addition, activated microglia can synthesize a range of different molecules, including free radicals, inflammatory cytokines, chemokines, lipid mediators, and neurotrophic factors, whose typical profile will determine the outcome of microglial activation in term of repair or injury [70]. Although in the past activated microglia have been regarded mainly as detrimental for the surrounding cells and as major players in neurodegenerative processes, it is now clear that activated microglia play complex and multifaceted roles, which need to be defined within each disease. Importantly, the different states of activation can be switched between one state and another during the course of disease or in response to further stimuli or signals from the periphery [72].

A deeper knowledge of microglial biology and of the molecular mechanisms underlying the acquisition of protective versus detrimental functions is crucial for finding new molecular targets and developing effective treatments for a wide range of neurological disorders.

In this view, PPAR-*γ* agonists have been extensively studied in the last decade for their therapeutic potential as key molecules in preventing the undesired toxic effects of microglial activation [35, 73].

One of the first finding supporting a role for 15d-PGJ_2_ as endogenous regulator of microglial activation—15d-PGJ_2_ derives from PGJ_2_, a major PG synthesized within the brain by most neural cells—was provided by Petrova et al. [74], who demonstrated that this PPAR-*γ* natural ligand attenuates iNOS expression, and the subsequent NO accumulation, in the murine BV-2 microglial cell line stimulated by LPS. Since the TZD troglitazone did not affect the NO pathway, it was suggested that 15d-PGJ_2_ inhibits iNOS expression by a PPAR-*γ* independent mechanism. The same authors then demonstrated that 15d-PGJ_2_ decreases the production of TNF-*α*, IL-1*β* and the expression of COX-2 in the same cell system while increasing the expression of the antioxidant enzyme hemeoxygenase-1 and the intracellular levels of glutathione [75].

Bernardo et al. [76] showed for the first time that primary microglial cells, unlike BV-2 cells, express PPAR-*γ* and that such basal expression is increased by its specific agonists, while it is reduced in the presence of microglial activators such as LPS and IFN-*γ*. Microglial PPAR-*γ* was subsequently shown to be functionally active, being able to bind specific PPRE sequences upon activation by natural and synthetic agonists [50]. Similar to BV-2 cell line, in primary microglial cultures 15d-PGJ_2_ prevented LPS-induced iNOS expression and TNF-*α* production as well as IFN-*γ*-induced expression of major histocompatibility complex (MHC) class II antigens, by mechanisms involving PPAR-*γ* activation and reduced activation of STAT-1 and NF-*κ*B, which are known to mediate IFN-*γ* and LPS signaling [76]. In human microglial cells, 15d-PGJ_2_ did not affect NF-*κ*B binding activity although it decreased STAT-1 expression and enhanced expression and binding activity of the AP-1 proteins J-Jun and c-Fos [60]. It was then reported that 15d-PGJ_2_ inhibits IL-12 synthesis in rat primary microglia and mouse cell line N9, activated either by LPS alone or in combination with IFN-*γ* or TNF-*α* [63, 77]. 15d-PGJ_2_ attenuated microglial activation also when elicited by Gram-positive bacteria Staphylococcus aureus, by inhibiting the expression of proinflammatory cytokines and the chemokine monocyte chemoattractant protein-1 (MCP-1) [73, 78].

In cortical mixed neuron-glial cultures 15d-PGJ_2_, ciglitazone and troglitazone prevented LPS-induced neuronal death, suggesting a PPAR-*γ* mediated mechanism of neuroprotection [79]. Similarly, 15d-PGJ_2_, ciglitazone, troglitazone and two NSAIDs indomethacin and ibuprofen reduced the neurotoxicity of microglial cells exposed to *β*-amyloid fibrils [80]. In this cell system, COX-2-specific inhibitors failed to promote neuronal survival, suggesting protective mechanisms independent of COX-2 metabolism.

In addition to indomethacin and ibuprofen, we have reported that two NO-releasing derivative of flurbiprofen, HCT1026 and NXC 2216, were able to prevent microglial activation by activating PPAR-*γ* [36, 37]. Interestingly, NCX 2216 after an initial activation induced PPAR-*γ* nitration and inactivation. These effects were paralleled by a transient reduction of TNF-*α* and NO production and a protracted inhibition of IL-1*β* and PGE_2_ synthesis, suggesting a dynamic regulation of the functional state of activated microglia by NCX 2216. Long treatment with NCX 2216 could therefore lead, after an initial activation of PPAR-*γ*, to a protracted suppression of its control over microglial activation. Our results could help explaining why among the few NSAIDs with A*β*-lowering activity (reviewed by [81]), only in the case of protracted administration of NCX 2216 in an AD animal model, the reduction of cerebral amyloid load accompanied by a sustained microglial activation [82].

The contribution of PPAR-*γ*-dependent or independent mechanisms to the inhibition of microglial activation by 15d-PGJ_2_ seems dependent on the cell type (primary versus transformed cell lines; fetal versus neonatal), or on concentration of the ligand. In rat primary microglial cultures, we have shown [50] that 15d-PGJ_2_ at concentrations several fold lower than those required for PPAR-*γ* activation, effectively reduced the LPS-stimulated production of PGJ_2_ by directly preventing the enzymatic activity of COX-2 rather than its expression, as previously described in activated monocytic cell lines [80, 83] and in BV-2 cells [75]. The reduction of COX-2 enzymatic activity could be achieved through the modifications of key cysteine residues [84], as suggested by the ability of 15d-PGJ_2_ electrophilic *α*,*β*-unsaturated ketones to modify several cellular proteins [85, 86]. At concentration 10 times higher than those required to activate PPAR-*γ*, 15d-PGJ_2_ induced microglial cell impairment and death by apoptosis [50]. The effects were stronger in activated microglia than in unstimulated cells, suggesting that this agent may prevent excessive microglial activation by promoting their elimination by apoptosis thus contributing to the resolution of inflammation as previously suggested in peripheral tissues [87, 88].

Although apoptosis by 15d-PGJ_2_ has been shown in several cells, the link between the proapoptotic effect of 15d-PGJ_2_ and PPAR-*γ* activation is still controversial. As before this may be linked to cell types and their degree of differentiation or transformation. For example, as opposed to the observations reported in primary microglia, the induction of apoptosis in T-cells and human and rat glioma cell lines appears mediated by PPAR-*γ*-dependent mechanisms [61, 87, 89–91].

## 6. CONCLUSIONS

In the last decade, there has been an increasing number of experimental studies supporting the use of PPAR-*γ* ligands to treat major disabling brain diseases, with a high social burden and impact on health case system. The compelling evidence obtained in experimental studies is complemented by sparse, but very encouraging clinical studies. The positive outcomes in animal models of AD, due to the ability of PPAR-*γ* agonists to reduced inflammation and the amyloid burden by various mechanisms, have found some validation in a pilot clinical trial in which AD patients treated for 6 months with rosiglitazone showed reduced attention and memory deficits [7]. In a second recent trial, the improvement in cognition after 6 months of rosiglitazone treatment was significant only in AD patients who did not have the *ε*4 allele of the apolipoprotein E [92], a genotype associate with a higher risk to develop AD. Similarly, the better neurological outcome reported after administration of PPAR-*γ* ligands in experimental stroke models are consistent with the result of a small clinical trial reporting that patients with diabetes receiving pioglitazone or rosiglitazone had an improved functional recovery after stroke compare to patients, who have not used any TZD [93]. Furthermore, a large clinical trial has demonstrated that pioglitazone reduced the combined risk of heart attack, stroke, and death in high risk type 2 diabetes patients [94].

The clinical use of PPAR-*γ* agonists in MS and ASL remains poorly investigated. Nonetheless, in a case report, pioglitazone treatment of an MS patient resulted in increased body weight and improved motor strength and coordination [27]. A first clinical trial for the use of pioglitazone in ALS started in Germany in late 2007.

Although PPAR-*γ* synthetic ligands such as TZDs and NSAIDs appear to be very promising drugs to treat severe human diseases, from cancer to metabolic diseases to brain diseases, several open issues still need to be examined. Among these, the toxic effect associated with some PPAR-*γ* agonists and their blood-brain-barrier permeability, which are at present still matter of controversies. A deep knowledge of the molecular mechanisms evoked by PPAR-*γ* ligands either dependent or independent of the receptor activation and of the dependence of such effects on the specific cell type is mandatory for the development of PPAR-*γ* drugs with increasing efficacy and safety.

## ABBREVIATIONS

15d-PJ_2_ =: 15-deoxy-Δ^12,14^-prostaglandin J_2_
AD=: Alzheimer disease
ALS=: Amyotrophic lateral sclerosis
AP-1=: Activator protein-1
CNS=: Central nervous system
COX=: Cyclooxygenase
DBD=: DNA-binding domain
EAE=: Experimental autoimmune encephalomyelitis
HODE=: Hydroxy octadecadienoic acids
IFN=: Interferon
IL=: Interleukin
iNOS=: Inducible nitric oxide synthase
JAK=: Janus activated kinases
LBD=: Ligand-binding domain
LPS=: Lipopolysaccharide
MAPK=: Mitogen-activated protein kinase
MCP-1=: Monocyte chemoattractrant protein-1
MHC=: Major histocompatibility complex
MS=: Multiple sclerosis
NF*κ*B=: Nuclear factor *κ*B
NO=: Nitric oxide
NSAIDs=: Nonsteroidal anti-inflammatory drugs
oxLDL=: Oxidized low-density lipoprotein
PD=: Parkinson disease
PPAR=: Peroxisome proliferator-activated receptor
PPRE=: Peroxisome proliferator response Elements

RXR=: Retinoid X-receptor
SOCS=: Suppressor of cytokine signalling
STAT=: Signal transducer and activators of transcription
Th=: T helper cell
TNF=: Tumour necrosis factor
TZDs=: Thiazolidinediones
